# D-Lactic Acidosis: An Underrecognized Complication of Short Bowel Syndrome

**DOI:** 10.1155/2015/476215

**Published:** 2015-04-22

**Authors:** N. Gurukripa Kowlgi, Lovely Chhabra

**Affiliations:** Department of Medicine, Hartford Hospital, University of Connecticut School of Medicine, Hartford, CT 06102, USA

## Abstract

D-lactic acidosis or D-lactate encephalopathy is a rare condition that occurs primarily in individuals who have a history of short bowel syndrome. The unabsorbed carbohydrates act as a substrate for colonic bacteria to form D-lactic acid among other organic acids. The acidic pH generated as a result of D-lactate production further propagates production of D-lactic acid, hence giving rise to a vicious cycle. D-lactic acid accumulation in the blood can cause neurologic symptoms such as delirium, ataxia, and slurred speech. Diagnosis is made by a combination of clinical and laboratory data including special assays for D-lactate. Treatment includes correcting the acidosis and decreasing substrate for D-lactate such as carbohydrates in meals. In addition, antibiotics can be used to clear colonic flora. Although newer techniques for diagnosis and treatment are being developed, clinical diagnosis still holds paramount importance, as there can be many confounders in the diagnosis as will be discussed subsequently.

## 1. Introduction

D-lactic acidosis (D-la) is a rare form of lactic acidosis seen mostly in patients with short bowel syndrome (SBS). Other conditions implicated are toxic ingestions of chemicals such as propylene glycol and rarely in patients with severe diabetic ketoacidosis. D-lactic acid is an enantiomer of L-lactic acid. L-lactic acid is the main entity involved in lactic acidosis in humans. Historically, D-la has been more commonly described in ruminants, with infrequent occurrence in humans [1]. The condition was first described in 1979 by Oh et al. [2] in a patient with SBS afflicted with severe metabolic acidosis and encephalopathy. SBS is a result of extensive surgical resection of the intestine, leading to malabsorption-induced diarrhea, electrolyte disturbances, and malnutrition due to poor nutrient processing [3]. The incidence of SBS can vary geographically, but the overall estimated incidence is approximately 2 persons per million per year [4].

The predominant organ system affected by D-la is the central nervous system (CNS). Presenting symptoms may include slurred speech, ataxia, altered mental status, psychosis, or even coma [2, 5–10]. A high index of clinical suspicion is the key in diagnosing D-la in patients with elevated anion gap metabolic acidosis, with normal L-lactate levels, history of SBS, and the above-described clinical features. This paper includes a comprehensive review of the etiology, clinical presentation, diagnostic techniques, and treatment modalities of D-la in patients with SBS.

## 2. Materials and Methods

In the preparation for this paper, we performed a PubMed and MEDLINE search of the published work in English language to identify case reports, review articles, research studies, and meta-analyses, on D-la in relation to SBS. Key words used were, “D-lact∗,” “malabsorp∗,” “short bowel,” “short gut,” “bowel surg∗,” “bowel resect∗,” “colectomy,” “ileal resect∗,” “Colon resect∗,” “colonic resec∗,” “ileal surg∗,” “colon surg∗,” “gut surg∗,” and “bariatric surg∗” which yielded 91 articles. Papers from January 1, 1979, to September 30, 2014, were included. Articles that fulfilled the criteria for adequate information including patient characteristics, presence of SBS, pathogenesis, clinical features, and management options of D-la were reviewed. Selected references from the original set of articles were included in the bibliography. For this paper, we selected 32 articles, based on the aforementioned criteria, which were relevant to our current discussion.

## 3. Objectives

This article aims to provide a comprehensive review of D-la utilizing the reported data in the literature and urges readers to recognize D-la in appropriate clinical settings. We also discuss in detail the diagnostic and therapeutic principles which aim to prevent the untoward consequences of D-la.

## 4. Pathophysiology of D-Lactic Acidosis

A Swedish chemist named Scheele is credited with the discovery of 2-hydroxypropanoate or lactic acid from sour milk [11]. Lactic acid exists as two enantiomers/stereoisomers, based on the orientation around its asymmetric second carbon atom. These are D (dextrorotatory) and L (levorotatory) types, based on the rotation of light in clockwise and counter-clockwise directions, respectively [12]. Normally, lactic acid exists entirely in L-lactate form (with a concentration of 1-2 mmol/L) as mammalian cells almost exclusively produce this form. The reason for this is the lack of the enzyme D-lactic acid dehydrogenase [13]. However, D-lactate can be formed in miniscule concentrations from methylglyoxal via the glyoxalase pathway [14].

Some fermented foods and beverages such as pickles, yogurt, sour milk, tomatoes, apples, beer, and wine can also increase D-lactic acid [15, 16]. The aforementioned mechanism is relevant to our discussion of D-la in patients with SBS. Compounds such as ringer lactate solution, sodium lactate, propylene glycol, and some peritoneal dialysate solutions can also cause D-la [14, 17]. Notably, both D and L-lactate can be produced by the microbiota in the gastrointestinal tract and the sole determinant of this is the amount of D or L-lactate dehydrogenase present in the bacterial flora of an individual [18]. Varying amounts of D and L-lactate can be formed depending on the relative concentration and biological activity of different bacterial species. Additionally, some bacteria have an enzyme DL-lactate racemase, which can convert one enantiomer to the other [18]. Originally, it was believed that due to the lack of enzyme D-lactate dehydrogenase in humans, we could not metabolize D-lactate to pyruvate. However, in the past two decades there is an abundance of literature suggesting the presence of an enzyme D-2-hydroxy acid dehydrogenase (D-2-HDH) that is mainly found in the liver and kidney [15, 17–22]. This enzyme is inhibited by low-pH states, which assumes importance in the relative overproduction of D-lactate in certain clinical situations and has been discussed in details later.

The production of D-lactate in the gut is precipitated by two factors: the first is the absence or reduced length of the small intestine which is normally responsible for the absorption of most simple carbohydrates; and the second is the presence of colon in continuity where all the unabsorbed carbohydrates are presented as a substrate for fermentation to the colonic bacterial flora. In healthy individuals, only dietary fiber, starch, and possibly a small amount of undigested mono- or disaccharides reach the colon where they are fermented and metabolized to organic acids. Usually, the rate of production of these organic acids does not exceed the rate of metabolism and hence, does not lead to clinically significant acidemia [23]. These organic acids mainly consist of acetate, propionate, and butyrate, collectively known as short-chain fatty acids (SCFA). These serve as the principal energy source of colonic mucosal cells, and even for the rest of the body at times. Fermentation of undigested carbohydrates eventually yields a very low concentration of D- and L-lactate [24] as they are metabolized to SCFA at a rate equaling their production [23].

Alteration of the colonic microbiota plays a major role in the production of D-lactate. Short bowel syndrome leads to an increased load of undigested carbohydrates (including simple sugars) in the colon. As a result, the amount of organic acid produced exceeds the amount that can be metabolized by healthy individuals. This leads to an accumulation of organic acids, including SCFA and lactate, resulting in a more acidic environment than normal. Interestingly, the lower pH favors the growth of bacteria responsible for producing D- and L-lactate as they are acid-resistant and this leads to a further decrease in the pH thus generating a vicious cycle. These bacteria include* Lactobacillus fermenti* and* L. acidophilus* amongst a few others [25, 26]. As mentioned previously, the primary enzyme responsible for metabolizing D-lactate in humans is D-2-HDH, which is inhibited in the acidic environment. Caldarini et al. [27] demonstrated this in an in vitro study performed on stool samples of a child. Thus, SBS leads to initiation and propagation of D-lactic acid formation.

The studies in infants have shown that, with development of SBS, it takes only 2-3 weeks of oral feeding to produce a dramatic change in their intestinal flora specifically lactate-producing lactobacillus, which increases from 1% to 60% [28, 29]. In spite of this dramatic change, it takes several months to years for the development of significant D-lactate in stool. Thus, it has been hypothesized that the establishment of new flora requires a significant length of time-period to take its effect [28]. Other conditions that can mimic SBS in producing the complications of D-la include inflammatory disease of the small bowel, especially Crohn's disease, antibiotics, and even probiotics that can alter the existing flora of the colon. Specific case reports are described in Table 1.

## 5. Review of Clinical Features of D-Lactic Acidosis

All cases of D-la described in this review had a history of SBS due to various causes including ischemic bowel, intestinal obstruction, congenital abnormalities, and midgut volvulus. There is an obvious domination of neurological symptoms in patients with D-la (Table 2). As evident from Tables 1 and 2, all patients exhibit some form of encephalopathy and this may pose a diagnostic challenge for clinicians since the presentation can often be confused with a primary neurological disorder. The primary neurological symptoms occurring in D-la are often attributed to acidosis. However, there have been reports refuting this causal association. Godey et al. [30] described a case of a 2-year-old female who was diagnosed with lactic acidosis and neurological symptoms. Patient had recurrences of neurological symptoms with elevated D-lactate but without significant acidosis 6 months from the original diagnosis.

In one of the first cases describing D-la in 1979, Oh et al. [2] proposed that the neurological manifestations are due to a direct toxic effect on the brain resulting from D-la crossing the blood-brain barrier. A possible mechanism suggested by the authors was that the brain tissue has lower levels of the D-lactate metabolizing enzyme D-2-HDH and, hence, the D-lactate levels rise. However, despite this theoretical basis, other case reports suggest that there is no difference in the concentration of D-lactic acid in blood or cerebrospinal fluid [31]. It is important to understand that the actual levels of D-lactate do not correlate with the clinical presentation. Experimental studies performed with loading of D-lactate in healthy patients using both intravenous and oral formulations, up to twice the upper limit of normal, resulted in only a few clinical symptoms [15, 21]. Thurn et al. [32] further strengthened this hypothesis in a study of 33 patients with history of jejunoileal bypass and also found a poor correlation between D-lactate levels and clinical symptoms.

Due to the aforementioned theory, it was thought that the neurological symptoms might not be related to D-lactate but to the other organic acids produced in the colon, which could be potentially neurotoxic. These include mercaptans, aldehydes, amines, and alcohols, which can potentially act as false neurotransmitters and give rise to clinical symptoms [23].

Other possible causes could be nutritional deficiencies as patients with SBS can lose significant absorptive surface area of the gut. Hudson et al. [8] reported a case of recurrent encephalopathy secondary to D-la in a patient who was found to have elevated erythrocyte transketolase levels suggestive of thiamine deficiency. The cerebellum is a potential target for damage in D-la. This is because it has a limited supply of pyruvate dehydrogenase, the enzyme required to convert pyruvate to acetyl co-A [26]. However, as D-lactate converts to pyruvate [33], the enzyme levels are not sufficient to metabolize all the pyruvate and this, compounded with a relative thiamine deficiency, may produce neurologic symptoms akin Wernicke's encephalopathy (confusion, ataxia, and ophthalmoplegia). Other theories include decreasing activity of pyruvate dehydrogenase with D-lactate accumulation due to a reduction in the pH [33]. While the pyruvate theory explains many of the clinical manifestations, there has been a case report of D-la where pyruvate levels were normal during acute neurological symptoms [30].

We previously discussed how SCFA are produced in the colon and serve as energy source to the colonic mucosal cells and sometimes even to the rest of the body. In cases of SBS, due to a change in microbiota, the lactate-producing bacteria tend to outgrow the ones producing SCFA. As a result, there is less acetyl-coA produced, leading to less ATP, which could affect neurotransmitter production and neurological functioning [33].

From the above discussion, it is clear that the manner in which D-lactate exerts its clinical influence is complex and there appear to be multiple factors that contribute to the outcomes. Nonetheless, this understanding of the pathophysiological mechanisms should prompt a physician to identify D-la in order to implement appropriate and timely therapeutic strategies.

## 6. Diagnosis of D-Lactic Acidosis

Diagnosis of D-la needs a high clinical suspicion and awareness of the different presentations of the condition, especially in patients with appropriate risk factors. The diagnosis should later be confirmed with appropriate lab testing. Common symptoms are delirium/encephalopathy, slurred speech, and unstable gait amongst others (see Table 1). Some of the cases described violent behavior while others suggested a presentation similar to acute alcohol intoxication. D-la should always be suspected in a patient with SBS, inflammatory bowel disease, or other malabsorption syndromes and it typically requires the presence of a relatively intact colon. Due to the nonspecific nature of symptoms, D-la is frequently missed [34] and, hence, a high index of suspicion is warranted in the above-mentioned patient population. Recent antibiotics or even probiotics have also been implicated in a few case reports [9, 35].

Blood work typically reveals anion gap metabolic acidosis, and if delta-delta ratio is calculated, it will range between one and two unless there are other additional contributing factors to metabolic acidosis. The rise in anion gap is less than expected. This is due to the stereo specificity of D-lactate, which makes it easier for D- rather than L-lactate to be excreted in urine. It can cause both an anion gap metabolic acidosis and a hyperchloremic metabolic acidosis. Sometimes the urine anion gap may be positive, and both this and hyperchloremic metabolic acidosis may lead to a false diagnosis of renal tubular acidosis (RTA) [34]. Urine osmolar gap can be used to calculate unmeasured cations such as NH4+, which is excreted more in D-la due to systemic acidosis and can help in differentiating from RTA. Special assays are required to measure levels of D-lactate as these assays contain D-lactate dehydrogenase. During acute episodes, the levels can rise and while there is no standard, a D-lactate concentration of >3 mmol/L is used to define D-la [36].

The symptoms of D-la are often transient making a biochemical diagnosis challenging. However, clinical history such as that described above should not be ignored. Patients may often give a history of prior neurological symptoms following consumption of a meal rich in carbohydrates. In some cases, physicians have challenged their patients to an enteral carbohydrate load for reproducibility of neurological symptoms and a rise in D-lactate levels [34, 37]. Electroencephalogram is infrequently utilized, as it is nonspecific. However, in an acute state, EEG abnormalities may coincide with D-lactate levels [38]. Stool cultures that grow high concentrations of D-lactate producing bacteria indicate D-la as a cause as well.

Henry et al. [39] recently studied novel methods of determining D- and L-lactate levels in the urine using high-performance liquid chromatograph-tandem mass spectrometry. The limit of detection of D-lactate was 0.125 mmol/L. More recent studies suggest that the ratio of D-lactate/L-lactate may serve as a better metabolic signature of altered colonic microbiota, as compared to the absolute values, and thus may help in a more reliable prediction of poor clinical outcomes in patients with SBS [40].

## 7. Treatment of D-Lactic Acidosis

Early identification and correction of metabolic abnormalities result in an improvement in the neurological symptoms. The acuity of treatment depends on the clinical status of the patient. The treatment plan can be summed up as follows.

1. Correct acidemia.
2. Removing the offending agent (carbohydrates, propylene glycol, or exogenous D-lactate).
3. Long-term control measures.

Intravenous bicarbonate can be used with fluid hydration to correct acidemia. Lactated ringer should be avoided as it contains both D- and L-lactate. During an acute episode, enteral carbohydrates should be avoided. Not only will this decrease the D-lactate due to lack of substrate but also the lactate-producing bacteria will starve and die in the absence of a food source [29]. Carbohydrates can be supplemented parenterally and thiamine supplementation should be considered to cover for the additional pyruvate dehydrogenase activity, especially in the cerebellum. Oral antibiotics that are poorly absorbed are most effectively used locally in the gut—these include clindamycin, vancomycin, neomycin, and kanamycin [33]. In rare cases, hemodialysis has been used to rapidly clear D-lactate from the plasma [41].

Once the acute phase is controlled, strategies for preventing future occurrences must be implemented. Long-term management should focus on avoiding the substrates responsible for D-lactate production. Simple carbohydrate restriction may be useful as they are metabolized to D-lactate more rapidly [18]. Some exogenous fermented foods such as sour milk, yogurt, and pickles have D-lactate and should be avoided.

Patients with SBS have fat malabsorption and this can lead to calcium soap formation in the gut with free oxalate being absorbed with the risk of renal stones. Patients should stay well hydrated to keep up with the losses. Also oxalate can inhibit D-2-HDH, and hence oxalate intake should be limited. Small amounts of calcium supplementation (up to 1 g/day) may be beneficial. This will also increase the pH in the bowel, which could decrease D-lactate production as shown by Caldarini et al. [27]. Long-term antibiotics can be considered in patients who have repeated episodes. However, with no standard guidelines, this should be on a case-by-case basis. Evidence of probiotics in D-la is unclear and controversial. There have been reports as described above regarding probiotics being implicated as a causative agent in a few cases of D-la [42] but there are no current recommendations to avoid them.

In those rare cases, where neither dietary nor medical treatments are effective, some surgical options might be beneficial. In SBS, certain procedures include intestinal lengthening [18], small bowel transplant, or, in some extreme cases, colon removal. There are some promising novel avenues emerging for the treatment of patients with SBS. One such modality in the experimental phase is technology of scaffolding. This technique involves production of tissue engineered intestine (TEI) using nanoparticles, which can take up the function of the intestine and help prevent the complications of SBS [43]. Further studies are needed in this field before it can be used safely and effectively in humans.

## 8. Future Direction

D-lactic acidosis is a rare condition with adverse clinical effects fortunately manageable with safe and effective treatment strategies. One aspect aiding the diagnosis of D-la could be the role of measuring organic acids produced during pyruvate metabolism. Further research is needed to better understand the pathophysiology of the associated neurological symptoms to address our knowledge gaps on this important issue.

## 9. Conclusion

Although D-la is a rare entity, it can negatively impact the quality of life in patients with SBS. Physicians should be aware of the risk factors and presenting symptoms while keeping a high-index of clinical suspicion, as early detection and treatment are imperative in order to effectively manage patients with this condition.

## Figures and Tables

**Table 1:** Case reports of D-lactic acidosis with short bowel syndrome.

Author/year	Patients	Age/sex	Cause of D-la	Comments/clinical
Kholostova et al./2014 [44]	24	<2 yrs./variable	SBS—ileojejunal rectal anastomosis for treatment of Hirschsprung's disease	Fecal D-lactate increased in 35% pts.
Singh et al./2013 [45]	1	37/M	Antibiotic induced alteration of colonic flora causing recurrent D-la	Complicated with new acute transient systolic CHF
Dahlquist et al./1984 [37]	1	24/F	SBS—ischemic small bowel resection	Primarily CNS symptoms-lethargy, echolalia, ataxia
Burski et al./2013 [46]	1	41/F	SBS—bariatric surgery	Confusion (feeling drunk), slurred speech, ataxia.
Gigante et al./2012 [47]	1	51/F	SBS—partial small bowel resection, jejunoileal bypass due to *Salmonella enteritidis* infection	Confusion, blurred vision, slurred speech, nausea, weakness
Guerrero et al./2010 [48]	1	35/F	SBS—ileal resection due to ischemic bowel	Dizziness, gait instability, loss of strength
Munakata et al./2010 [42]	1	5/F	SBS—jejunoileal atresia; ileus; peritonitis probiotic use—*Bifidobacterium *	Intermittent ataxia, inability to grasp objects, explosive speech
Grunert et al./2010 [49]	1	7/M	Congenital disaccharidase deficiency	Found comatose after parenteral nutrition was held, and pt. had oral meals, slurred speech
Dahhak et al./2008 [50]	1	6/M	SBS—bowel surgery indication not reported	Dysarthria and disorientation
Soler Palacín et al./2006 [51]	1	11/M	SBS—multiple bowel resections in the postnatal period	Confusion, encephalopathy, slurred speech
Puwanant et al./2005 [52]	1	14/M	SBS—midgut volvulus surgery five years prior	Episodic confusion, hyperpnea
Uchida et al./2004 [53]	1	22/M	SBS—volvulus surgery at age three	Slurred speech, somnolence, gait disturbance
Zhang et al./2003 [16]	1	12/M	SBS—20 cm resection of small bowel due to volvulus	11 episodes of weakness, ataxia, nausea, slurred speech, lethargy
Kamar et al./2003 [54]	1	80/M	SBS—subtotal colectomy with ileal J-pouch	Confusion, disorientation, tachypnea
Azhar and Beach/2002 [55]	1	53/F	SBS—colectomy and colostomy 20 years prior to Ulcerative colitis	Nausea, vomiting, fatigue, diarrhea from colostomy
Angelet et al./2002 [56]	1	Unknown	SBS	Neurological symptoms
Lalive et al./2001 [57]	1	54/—	SBS—extensive small bowel surgery few months prior	Several acute confusional episodes, ataxia, nystagmus, dysarthria
Sela et al./1999 [58]	2	2.25/F 1.5/M	SBS—congenital intestinal obstruction	Encephalopathy, ataxia
Day and Abbott/1999 [5]	1	Unknown	SBS	Unknown
Vella and Farrugia/1998 [33]	1	50/F	SBS	Recurrent weakness, ataxia, slurred speech, confusion, nausea
Coronado et al./1995 [9]	1	50/M	SBS—Jejunoileal bypass 2 years prior, with recent doxycycline use	Ataxia, slurred speech, weakness
Koletzko et al./1994 [59]	1	9/M	SBS—small intestinal duplication 2 years prior	Recurrent episodes—failure to thrive, unsteady gait, slurred speech, somnolence
Gurevitch et al./1993 [60]	1	1.6 yrs.	SBS—midgut volvulus	Encephalopathy syndrome
Forsyth et al./1991 [61]	1	Unknown	SBS—jejunal atresia	Recurrent encephalopathy, treated with neomycin
Flourie et al./1990 [35]	1	24/M	SBS—recent treatment with Bactrim	Neurological symptoms
Scully et al./1989 [31]	1	16/M	SBS—small bowel ischemia from stab wounds	Headache, confusion, stupor, dysarthria
Rosenthal and Pesce/1985 [62]	1	2.5/M	SBS—midgut volvulus	Ataxia, lethargy
Haan et al./1985 [63]	1	8/M	SBS	Depression, slurred speech, confusion, aggressive behavior intermittently
Traube et al./1983 [64]	1	40/M	SBS—jejunoileal bypass	Bizarre behavior, slurred speech, ataxic gait
Satoh et al./1982 [65]	2	Unknown	SBS	Unknown
Schoorel et al./1980 [66]	1	3/M	SBS	Confusion, dyspnea, drowsiness
Oh et al./1979 [2]	1	30/M	SBS—ischemic bowel	Slurred speech, confusion

**Table 2:** Prevalence of symptoms with D-lactic acidosis in patients with short bowel syndrome.

Presentation	Percentage of patients
Encephalopathy	100
Slurred speech	52
Ataxia	32
Gait disturbance	29
Weakness	16
Tachypnea	13
Aggressive behavior	10
Diarrhea	6
Acute CHF	3
Headache	3
Nystagmus	3
Blurry vision	3
Explosive speech	3
Echolalia	3
“Feeling drunk”	3
Depression	3
